# To Be or Not IIb: A Multi-Step Process for Epstein-Barr Virus Latency Establishment and Consequences for B Cell Tumorigenesis

**DOI:** 10.1371/journal.ppat.1004656

**Published:** 2015-03-19

**Authors:** Alexander M. Price, Micah A. Luftig

**Affiliations:** Department of Molecular Genetics and Microbiology, Duke Center for Virology, Duke University Medical Center, Durham, North Carolina, United States of America; University of Florida, UNITED STATES

Epstein-Barr virus (EBV), a γ-herpesvirus, was the first human tumor virus discovered fifty years ago in endemic Burkitt lymphoma [1]. Surprisingly, EBV was subsequently recognized to be a ubiquitous virus infecting greater than 90% of adults worldwide. In healthy children, primary EBV infection is typically asymptomatic because of a robust T cell immune response. However, infection during adolescence or later can result in infectious mononucleosis. Regardless of the severity of primary infection, EBV establishes a lifelong latent infection in the peripheral blood memory B cell compartment [2]. Immune suppression, genetic predisposition, or environmental factors can all serve to promote EBV-driven tumors, which are primarily of B cell, but also of epithelial and NK or T cell origin.

Early studies demonstrated that EBV infection in vitro transformed resting B cells into immortalized lymphoblastoid cell lines (LCL), providing a strong link to EBV-associated B cell cancers [3,4]. These pioneering studies stimulated intense investigation over the ensuing decades into the viral requirements and temporal cascade of gene expression triggering physiological cell changes associated with B cell proliferation and survival. The model that emerged indicates that B cell transformation in vitro is a latent infection including expression of a discrete set of nine proteins and many noncoding RNAs collectively called Latency III [2]. In this model, all of the Latency III gene products are expressed prior to the first cell division in the absence of lytic virus replication. However, this in vitro model does not account for the viral gene expression programs found in most EBV-positive B cell tumors or in the B cell compartment in vivo following natural infection. Moreover, a recent resurgence in studies of the earliest events following B cell infection combined with an appreciation of the heterogeneity of EBV latent gene expression during natural infection and in tumors provides the rationale for revisiting how this ubiquitous virus establishes latency and causes B cell malignancies.

## Early B cell infection: Prelatency, the Epstein-Barr Nuclear Antigen (EBNA) transcriptional unit, and induction of proliferation

**Fig 1:**
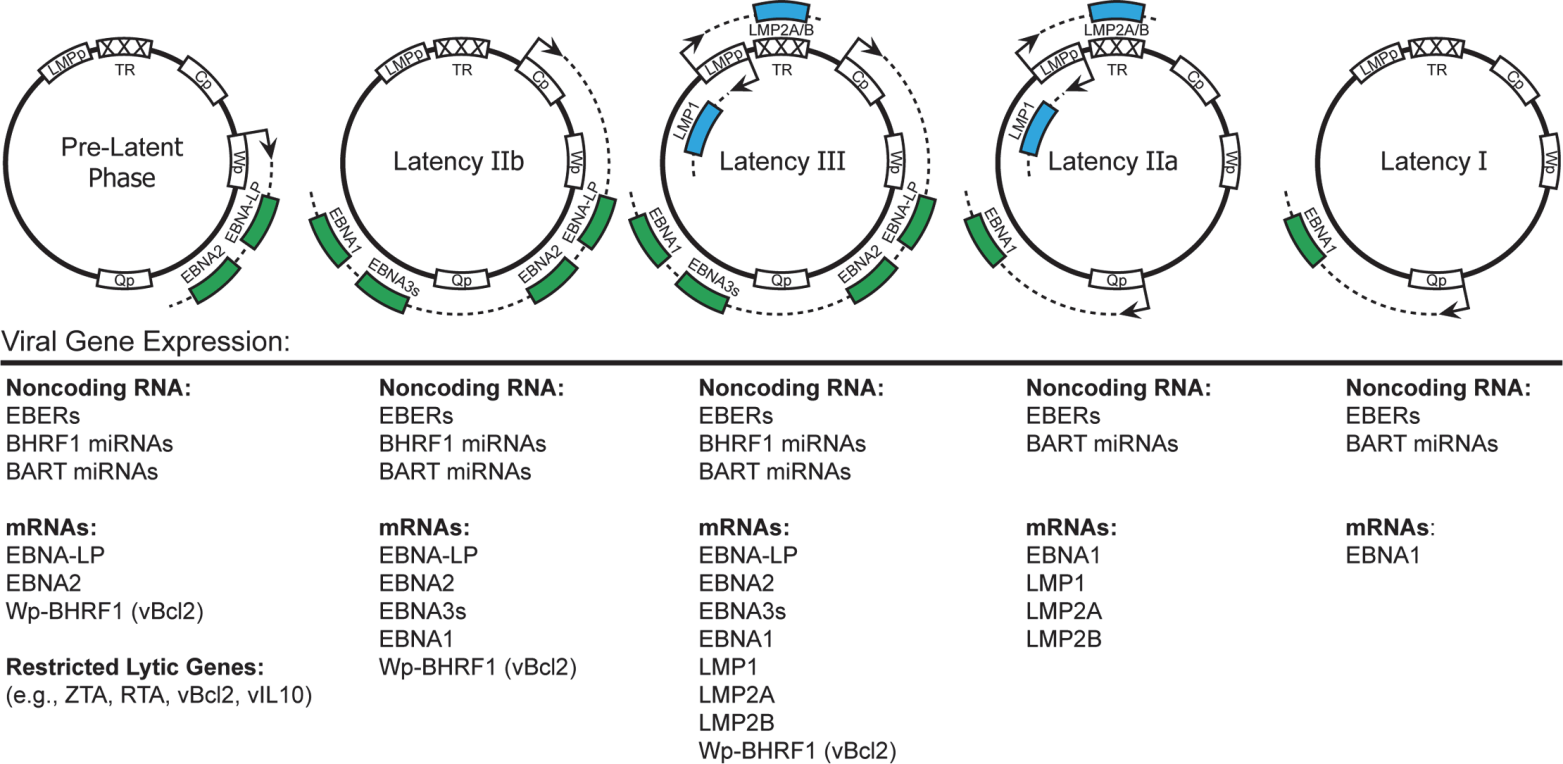
EBV latency gene expression in different latency states. This figure depicts, from left to right, the theoretical progression of EBV latency gene expression from initial infection to true latency. The EBV genome is shown in episomal form closed at the terminal repeats (TR). Promoters are shown as white boxes and include the EBNA promoters Cp, Wp, and Qp as well as the bidirectional LMPp. Primary mRNA transcripts are shown as dotted lines, while coding regions have been simplified as colored boxes. An expanded list of viral genes expressed in each latency state is listed directly underneath the representative schematic.

The initial events following EBV entry into B cells include deposition of the viral DNA into the nucleus and circularization of the linear genome followed by chromatinization and initial viral latent gene expression [2]. Recent work has refined these earlier studies to define a period after infection, but before cell division, of promiscuous viral gene expression where many lytic cycle mRNAs are produced in the absence of lytic DNA replication [5–8]. This transient burst of lytic gene expression is concomitant with transcription from the initial EBV latency promoter, the W promoter (Wp), to produce the first so-called latency associated proteins [9]. Together, this period of gene expression before cell division and up to the very first cell division is called the prelatent phase (Fig. 1). As the lytic mRNAs wane, the viral Epstein-Barr Nuclear Antigen 2 (EBNA2) and EBNA-LP proteins accumulate and directly activate the major upstream EBV latency C promoter (Cp) at ~48–72 hours postinfection [10]. Prior to the first cell division, at approximately 84 hours postinfection, the viral EBNA3A, 3B, 3C, and EBNA1 proteins are all expressed [10,11]. EBNA3s antagonize tumor suppressors while EBNA1 facilitates EBV genome replication and persistence. Concomitant with EBNA expression, viral BART and BHRF1 miRNAs are expressed as well as the pol III-driven short noncoding EBER RNAs [2]. Recently, it has become appreciated that the viral BCL-2 homolog, BHRF1, is expressed and peaks upon initial infection and is then continuously expressed at low levels from Wp through long-term outgrowth [12].

The first four cell divisions following infection are fast, on the order of 8–12 hours per cycle [11]. This rapid cycling mimics the behavior of centroblasts of the germinal center (GC). As a consequence of this rapid proliferation, EBV-infected blasts are susceptible to growth arrest due to a hyperproliferation-associated DNA damage response [11,13]. Infected cells that attenuate their proliferation rate will become immortalized in vitro. Interestingly, these early-infected cells express all of the EBNA proteins, but only minimally express the latent membrane proteins, or LMPs, 1, 2A, and 2B [14]. This cell state has been referred to as Latency IIb and may represent a clinically relevant feature of EBV-associated lymphomas as will be addressed below.

## Transition to Latency III: Expression of the viral EBNAs and LMPs in LCLs in vitro and lymphomas in the setting of immune suppression

The full Latency III viral gene expression program, also known as the growth program, is characterized by the expression of all of the EBNAs as well as the LMPs (Fig. 1). The least restrictive of all the latency states, Latency III is observed in LCLs generated in vitro and in lymphomas in the setting of immune suppression, such as post-transplant or HIV/AIDS [2]. Latency III cells are characterized by high activity of the host NFκB pathway, which is primarily imparted by the viral LMP1 protein, a constitutively active mimic of the host CD40 costimulatory molecule [15]. LCLs and Latency III-expressing EBV lymphomas depend on high levels of NFκB activity for their survival [16,17]. Given that LMP1-induced NFκB activity is low during the first two weeks after infection [8,14], how these early-infected cells survive is an important unresolved question in the field of EBV biology.

Despite the canonical role for LMP1 in promoting survival, high level expression of LMP1 and NFκB activity might be deleterious to infected lymphoblasts in vivo. In Burkitt lymphoma, it was discovered that high levels of c-Myc transcriptional activity and a lack of NFκB imposed a nonimmunogenic phenotype due to low levels of major histocompatibility complex (MHC) class I expression and antigen presentation [18]. Furthermore, high levels of MHC class I-restricted immunogenicity was restored when c-Myc was attenuated and the cellular growth phenotype was converted to Latency III (i.e., high NFκB) [18]. Consistent with this dichotomy, Latency III gene expression in EBV-infected cells in vivo is mostly observed in settings of T cell immune suppression such as following organ transplant or HIV/AIDS [2,17].

## Restricted EBV Latency IIa in germinal centers and Hodgkin lymphoma

While the progression of EBV latent infection in vitro culminates with a Latency III-expressing LCL, in vivo the establishment of latency is likely to proceed through a more complex route. It is known that EBV-infected cells express Latency III proteins at some point during natural infection, because EBV-specific cytotoxic T lymphocytes (CTLs) recognize immunodominant epitopes found in the EBNA3s [19]. Indeed, it is likely selective pressure from EBV-specific CTLs, in part, that promotes the transition from Latency III to more restricted forms of latency. Latency IIa has been detected in GC cells of healthy individuals as well as in EBV-positive Hodgkin lymphoma [20]. This form of latency is characterized by the lack of EBNA expression from Cp or Wp, retained expression of LMP1 and LMP2A, the expression of EBNA1 from a unique promoter (Qp), and the expression of EBER RNAs and BART miRNAs (Fig. 1). Precisely how the transition to this restricted latency state occurs is not well understood. However, recent work implicates T cell secreted cytokines, including IL-21, in down-regulation of Cp and EBNA expression [21].

Latency IIa-expressing cells are also thought to be precursors of EBV-associated Hodgkin lymphomas. These tumors often contain crippled immunoglobulin genes, which are likely to have been rescued through the mimicry of CD40 and B cell Receptor (BCR) signaling by LMP1 and LMP2A, respectively [22]. During natural infection, the LMP-mediated survival of GC B cells may promote entry into the mature memory pool in the periphery. However, whether antigen-driven BCR signaling is required for this process remains to be determined.

## True latency: Latency 0/I in resting memory B cells and Burkitt lymphoma

In vivo, the major pool of EBV latently-infected B cells are resting, peripheral blood class-switched memory cells [23]. No viral proteins are expressed in these cells (Latency 0) except during cell division, when EBNA1 is expressed from Qp (Latency I) (Fig. 1). Interestingly, EBV ncRNAs including EBERs and BART miRNAs are expressed in these cells [2]. Therefore, these cells are poorly recognized by the T cell immune response. It is unclear what regulates viral gene expression in these truly latent cells; perhaps the lack of proliferation-associated transcription factors prevents activation of the EBNA and LMP promoters. The post-GC ontogeny, poor immunogenicity, and restricted latent gene expression of these cells are also reflected in EBV-associated Burkitt lymphomas.

## The prevalence and newly appreciated importance of Latency IIb (EBNA+/LMP-) during latency establishment and in tumors

Latency IIb is a form of latency that was first observed following primary infection of B cells derived from patients with chronic lymphocytic leukemia [24]. It was named Latency IIb by Eva Klein because the EBNA2+/LMP1- gene expression phenotype is directly opposite of the EBNA2-/LMP1+ phenotype observed in Latency IIa [25]. Recent work has demonstrated that this latent gene expression state is also observed after EBV infects peripheral blood B cells and lasts for approximately two weeks before transitioning to the full Latency III state found in LCLs [14].

**Fig 2:**
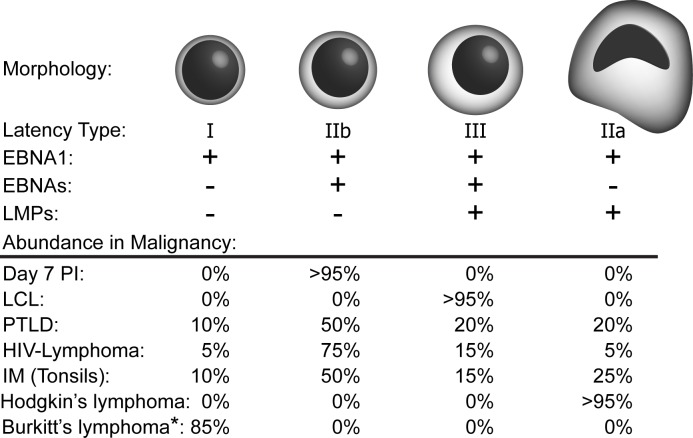
EBV latency types found in EBV-driven malignancies. Cell morphology (including cellular size and classic Reed–Sternberg-like nuclear morphology in the case of Latency IIa cells) that is associated with various EBV latency types is depicted. In heterogeneous malignancies, the relative percentage of cells displaying each latency type as shown by tumor cross-sections and immunohistochemistry are shown below (Relevant citations: Day 7 post infection [14], Post-Transplant Lymphoproliferative Disease (PTLD) [26,27], HIV-Lymphoma [26], IM [28], all others [2]). *Of note, approximately 15% of Burkitt lymphomas carry a mutant EBV genome containing a deleted EBNA2 gene, resulting in expression of the EBNA3s and Wp-BHRF1 (vBcl2), as well as EBNA1.

A subset of EBV-associated malignancies including HIV-associated lymphomas and post-transplant lymphomas display heterogeneous expression of Latency IIb in addition to Latency III [17,25–27]. These findings are based on single cell analysis of tumor sections using immunohistochemistry and contrast the dogma based upon bulk expression analysis that characterized these malignancies as Latency III [2]. These single cell data, coupled with the aforementioned finding of Latency IIb during early primary B cell infection suggests that this form of latency may contribute to EBV pathogenesis [14,25]. Indeed, heterogeneous expression of Latency IIb with other latency types was also observed in tonsils of acute infectious mononucleosis (IM) patients [28]. A summary of latency types observed in EBV-infected B cells in vitro and in vivo is presented as Fig. 2.

The prevalence of cells expressing Latency IIb within tumors is hypothesized to be due to the highly immunogenic state conferred by LMP1 within Latency III. Indeed, in humanized mouse model systems of EBV infection with functional adaptive responses, the ability of T cells to specifically kill EBV-infected B cells selects for Latency IIb-expressing tumors [29]. In recent cell line studies, Latency III-expressing LCLs were found to cycle through a wide range of expression of LMP1, which correlated with MHC I surface levels such that only cells expressing the highest amount of LMP1, and consequently MHC I, were efficiently killed by CTLs [30]. These data beg the important question of whether the LMP1–MHC I connection is also observed in vivo; will tumor cells displaying a Latency III expression pattern be killed by CTLs while those displaying a Latency IIb program be spared? A related question is whether CD4+ T cells or NK cells might play a more important role in controlling Latency IIb expressing cells in vivo. The answers to these questions will be critical for defining the mechanisms of EBV latency establishment and B cell tumorigenesis in the immunodeficient and the immunocompetent host.
